# Perception of Threatening Intention Modulates Brain Processes to Body Actions: Evidence From Event-Related Potentials

**DOI:** 10.3389/fpsyg.2018.02149

**Published:** 2018-11-27

**Authors:** Guan Wang, Pei Wang, Junlong Luo, Wenya Nan

**Affiliations:** ^1^School of Education Science, Huaiyin Normal University, Huaian, China; ^2^Department of Special Education, Faculty of Education, East China Normal University, Shanghai, China; ^3^Department of Psychology, Shanghai Normal University, Shanghai, China

**Keywords:** body action, threatening intention, early posterior positivity, reaction time, event related potentials

## Abstract

Efficiently perceiving a threatening intention conveyed by others’ bodily actions has great survival value. The current study examined if the human brain is sensitive to differences in intentions that are conveyed via bodily actions. For this purpose, a new intention categorization task was developed in which participants sat in front of a computer screen on which the pictures of highly threatening (HT), moderately threatening (MT), and non-threatening (NT) body actions were presented randomly. Participants were asked to press the corresponding buttons using threatening intention judgment, while event-related potentials (ERPs) were recorded. According to a cluster permutation test, we analyzed N190, N2, EPP (early posterior positivity), and P3. The results showed there was a positive correlation between the amplitude of the EPP induced by three kinds of body actions and the reaction time of the task. The results also revealed that when the deflection of EPP was less positive, the reaction time was shorter. We suggest that EPP might be useful as an index of body intention processing of the brain. The current study revealed that intention perception of body actions modulates brain processing.

## Introduction

The issue of threat detection was vital in many situations during human phylogenesis to allocate attention to threats to facilitate adequate reactions in due time (Feldmann-Wüstefeld et al., 2011). Threats are usually thoroughly processed (Williams et al., 2006), and they prepare the body for greater action tendency (Schutter et al., 2008; van Loon et al., 2010; Borgomaneri et al., 2014) and for quick actions (Coombes et al., 2005; Blanchard et al., 2011). This phenomenon has been shown to occur in response to threat-related words (Fox et al., 2001), pictures (Yiend and Mathews, 2001), faces, or movements (Fox et al., 2001, 2002; Borhani et al., 2015; de Valk et al., 2015). For example, anger compared with neutral body movements not only induced larger peak amplitudes on early body-related components, such as the N190 (Thierry et al., 2006; van Heijnsbergen et al., 2007; de Gelder et al., 2015), EPN (early posterior negativity) (Price et al., 2012; Borhani et al., 2015), but also boosted later potentials that reflected decision making processes (Liddell et al., 2004; Borhani et al., 2015). Furthermore, extensive studies on the processing of body movements showed that anger or fear body movements yielded automatic defense responses, such as fight, flight, and freeze (Tamietto and De Gelder, 2010; Pichon et al., 2012; de Gelder, 2013; Kveraga et al., 2015). Thus, humans could be well adapted to the quick recognition of another’s threat and could mount an adequate response to it.

The behavioral evidence from threat detection has found that anger, rather than negative emotion, triggers faster cognitive processes (Juth et al., 2005; Schimmack and Derryberry, 2005; Frischen et al., 2008). For example, schematic angry, happy, and neutral faces were used to test the hypothesis that humans preferentially orient their attention toward a threat, which showed that angry faces were more quickly and accurately detected than other negative emotion faces (sad or “scheming”) (Feldmann-Wüstefeld et al., 2011). Other non-verbal stimuli, such as body actions, convey information, and have also demonstrated this phenomenon. Gilbert et al. (2011) first adopted a visual search paradigm to explore attention bias toward body movements. The results showed that individuals detected the discrepant angry movement in a negative emotion crowd at a faster speed, whereas they detected the discrepant negative emotion movement in an angry crowd at a slower speed. The results also showed that the human brain had an attention-holding advantage of threat (Fox et al., 2001; Azarian et al., 2016). Thus, we inferred that other information, but not negative emotion signals, could explain the induced threat detection advantage.

Emotional body movements, such as anger or fear, have emotion-intention duality (i.e., emotion is intertwined with the intention in the emotional body movements) (Schupp et al., 2004; de Gelder, 2006; Azarian et al., 2016). Thus, the human brain not only processes emotional signals, but also processes the intention conveyed by such body movements (Heberlein, 2008; Steel et al., 2015; Okruszek, 2018). Considering the interference effect of prior and automatic processing of emotion relative to intention (Chouchourelou et al., 2006; Rellecke et al., 2012), it remains unclear whether an electrophysiological component is specifically responsible for processing threatening intention from body movements.

Few studies have directly explored the underlying neurophysiological characteristics of threatening intention processing, although the results of above behavioral experiments have partly conformed to this reasoning. In a neurophysiological study on intention (Schupp et al., 2004), participants were asked to observe human angry and fear faces in the absence of any task or instruction to evaluate the categorization of the expressions. The results showed that EPN bilaterally pronounced relative negativity over temporo-occipital areas, showing significant differences in different types of facial stimuli. Thus, the study suggested that the EPN represents enhanced processing of threat intention. However, in that study, there were at least two factors that need to be clarified. First, extensive studies have shown that the EPN reflects sensory encoding of emotional stimuli (Junghöfer et al., 2001; Uusberg et al., 2013; Borhani et al., 2015), and in the study of Schupp et al. (2004), the task did not require participants to identify emotion; however, EPN still emerged in the electroencephalogram (EEG). Thus, we speculated that the EPN could be an automatic process indicator of emotional stimuli, independent of task instruction. Our inference was also in line with two-stage models of visual perception that associated the EPN with initial relevance detection (Uusberg et al., 2013). This view also suggested that the EPN is not suitable to explore the brain sensitivity to intention conveyed by emotional body movements. Second, it is easy to overlook the results of the study of Schupp et al. (2004). The authors showed that compared with friendly and neutral faces, threatening faces induced a significant positive deflection in the time windows of 220–320 ms on centroparietal sites. Thus, we inferred that this component could represent intention processing, and we called this component EPP (early posterior positive). Based on the interaction effect of emotion with intention processing in the emotional body movements, and the importance of detecting intention for the conduct of social life, the current study was designed to investigate, using the high temporal resolution of ERPs, whether there was a special electrophysiological component responsible for the intention decoding of threatening body movements.

In addition, the current study is also the first to explore the behavioral characteristics induced by threatening body movements. Using a new experimental paradigm, this study investigated the brain processing toward highly threatening (HT), moderately threatening (MT), and non-threatening (NT) body movements, as played by two male actors. We opted for these actions for two reasons. First, in contrast to emotional body movements, threatening body movements only transmit explicit intention without transmitting any emotional signals. Second, male actors were recruited exclusively because previous research has shown they evoke fewer affective responses than female actors (Kret et al., 2011). This study aimed to investigate whether EPN could also be induced by threatening intention and if EPP could be used an index of intentional action processing. In summary, we expected that EPP would emerge as a special component in the brain processing stream that is sensitive to threatening intention.

## Materials and Methods

### Participants

Twenty-seven participants (15 male) took part in the experiment. The mean age was 22.21 [standard deviation (*SD*) = 2.36], with ages ranging from 18 to 24 years old. The participants were recruited at the psychology laboratory of the Shanghai Normal University, Shanghai, China. They filled out an informed consent form and were debriefed after the experiment, for which they obtained course credit. Participants had no neurological or psychiatric history, and were right-handed and had normal or corrected-to normal vision. The study was performed in accordance with the Declaration of Helsinki and approved by Shanghai Normal University ethical committee.

### Materials Construction

This study used experimental materials showing intentional actions instead of emotional body movements. There were three steps to ensure no explicit emotion in our intentional action pictures. At step 1, one semiprofessional male actor was hired and instructed to play various actions in three different scenarios (attacking another person, dancing with a lover, and harming others with a knife), which were recorded by a camera located at an angle of 90° relative to directly facing the actor. Finally, 30 pictures were required in which the figure faced another with the movements of stabbing (HT), fighting (MT), or dancing (NT) serving as the intentional actions. These stimuli were rated for threat on 7-point scales (1 = “high threat”; 7 = “low threat”) using an online sample [*N* = 91, 53 female, mean deviation of age (*MD*_age_) = 25, *SD* = 2.29, range = 19–35] on Wenjuanxing^1^. The results showed that stabbing actions were rated as more threatening (*M* = 1.60, *SD* = 0.28) than fighting (*M* = 2.22, *SD* = 0.43) [*t*(89) = 37.344, *p* < 0.001, *d*s = 5.95] and dancing (*M* = 5.12, *SD* = 0.38) [*t*(89) = 47.586, *p* < 0.001, *d*s = 7.65]; fighting actions were rated as more threatening than dancing [*t*(89) = 26.684, *p* < 0.001, *d*s = 3.75].

In step 2, we randomly chose 30 emotional movement pictures (fear, anger, and happy) from the BEAST stimulus database (de Gelder and Van den Stock, 2011) (Angry: F01AN, F02AN, F03AN, F04AN, F05AN, F06AN, F07AN, F08AN, F09AN, and F10AN; Fear: F01FE, F02FE, F03FE, F04FE, F05FE, F06FE, F07FE, F08FE, F09FE, and F10FE; Happy: F01HA, F02HA, F03HA, F04HA, F05HA, F06HA, F07HA, F08HA, F09HA, and F10HA). In step 3, we compared two sets of pictures to demonstrate that the pictures we made are different from the emotional pictures and have no emotional signals. All these pictures were mixed into a set and silhouetted using GIMP^2^. We then invited 92 participants to classify them according to emotion by pressing the corresponding key (1: angry, 2: disgust, 3: fear, 4: happy, 5: sad, respectively). The final results showed that the accuracy of identifying the emotion shown in the pictures obtained from the BEAST database was about 91% (angry), 95% (fear), and 96% (happy), and the mean reaction time was 510 ms (angry), 525 ms (fear), and 499 ms (happy). However, for intention pictures, the mean reaction time was 980 ms and the probability of HT pictures being categorized as anger, fear, sadness, happiness, or disgust were 33, 30, 23, 10, and 4%, respectively. The probability of the MT pictures being categorized as anger, fear, sadness, happiness, or disgust were 31, 32, 22, 12, and 3%, respectively. The probability of the NT pictures being categorized as anger, fear, sadness, happiness, or disgust were 25, 21, 10, 25, and 19%. Thus, the emotional categorization of the intentional actions occurred by chance, which showed that that intentional pictures did not convey any specific emotion. All of these results testified that intentional body movements from step 1 did not convey any explicit emotion.

### Subjective Rating of the Action Intention

Thereafter, the 30 intentional pictures (including 10 HT, 10 MT, and 10 NT) were equated for luminance and root mean square contrast (not including the gray background in calculation), and validated the threat intention of these body actions. Thirty students majoring in psychology evaluated the threatening intention (rated on 7-point scale, from 1 “the least threatening” to 7 “the most threatening”) and the friendly intention (rated on 7-point scale, from 1 “the least friendly” to 7 “the most friendly”) for the action pictures under all three conditions. Stimulus was presented in a pseudo random sequence, and Table 1 shows the results of all three conditions. One-way analysis of variance (ANOVA) showed that the main effect of different actions on threatening intention rating was significant, *F*(2,88) = 110.127, *p* < 0.001, $\eta_{p}^{2}$ = 0.227. More specifically, stabbing was perceived as the most threatening in all kinds of actions [*t*(29)_stabbing-fighting_ = 59.351, *p* < 0.001; *t*(29)_stabbing-dancing_ = 89.732, *p* < 0.001], fighting was perceived as more threatening than dancing actions [*t*(29)_fighting-dancing_ = 35.668, *p* < 0.001]. Meanwhile, the main effect of the different kind actions on the friendly intention rating was also significant, *F*(2,88) = 64.359, *p* < 0.001, $\eta_{p}^{2}$ = 0.239. Dancing actions, in which there was no significant difference between MT and HT, were perceived as friendlier than the other two kinds of actions [*t*(29)_dancing-fighting_ = 21.259, *p* < 0.001; *t*(29)_dancing-stabbing_ = 52.722, *p* < 0.001]. Fighting was perceived as friendlier than stabbing [*t*(29)_fighting-stabbing_ = 17.645, *p* < 0.001], which was perceived least the friendly of all the kinds of actions.

**Table 1:** Mean threat and friendliness ratings when view non-threatening, moderately, and highly threatening body actions.

Dependent	Dancing images (NT)		Fighting images (MT)		Stabbing images (HT)	
Measure	*M*	*SD*	*M*	*SD*	*M*	*SD*
Threatening rating (1–7)	1.72	1.01	5.01	2.45	6.84	1.16
Friendliness rating (1–7)	4.25	1.14	1.23	1.73	1.08	0.62

### Procedure

A new intention categorization task was conducted. Participants were seated in a darkened and sound-attenuated room, and received the presentation of stimuli on a 19′′ monitor (1024 pixels × 1024 pixels) with a gray background. All picture stimuli had the same size (10 cm × 10 cm). The viewing angle was set at about 9.5° × 9.5°, and the picture size was 512 pixels × 512 pixels, and the image sampling rate was 72 ppi. After participants read the information brochure and signed the informed consent, and then given verbal instructions. The distance between the participants and screen was 80 cm, a distance at which all participants could comfortably press the keyboard. HT, MT, and NT body actions were randomly presented on the computer screen using E-prime 2.0 (Psychology Software Tools, Inc., 2012). The participants pressed the “J” button with the index finger for threatening movements and the “L” button with the ring finger for NT movements.

**FIGURE 1:**
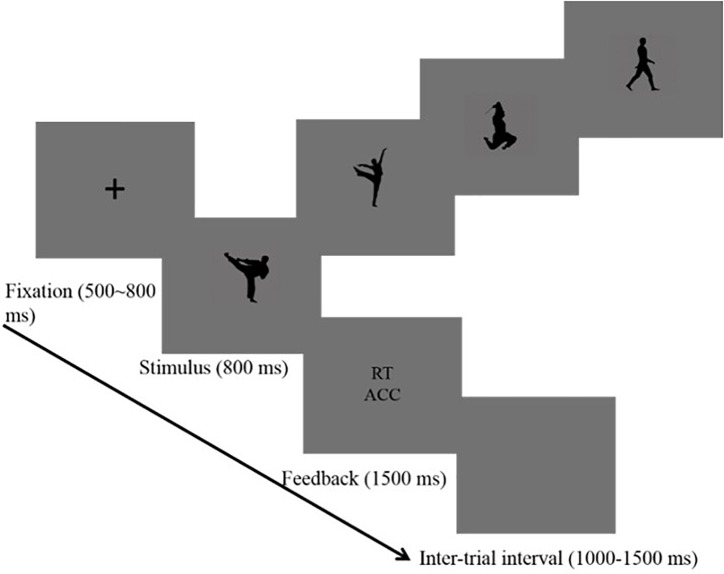
Procedure used in the categorization task that shows the sequence of events within a trial.

The trial started with the presentation of a fixation (“+”) in the center of the screen for 500–800 ms randomly. Second, a black-silhouetted body action appeared on the screen for 800 ms. In this phase, participants were instructed to press buttons. After that, a feedback screen displaying the reaction time and accuracy was sustained for 1500 ms, and the inter-trial interval was 1000–1500 ms randomly (see Figure 1). Participants were asked to focus on the central fixation point and to avoid head movement or eye blink as much as possible. Reaction times were measured and the EEG was recorded. There were eight blocks with a total of 480 trials using a random order. In each block, 60 trials were randomly presented (20 trials × 3 body stimuli: HT, MT, and NT). A self-paced break was provided at each block. The trial number in this study met the requirement that the number of trials in ERP studies should be high enough to reach a high signal-to-noise (SNR) (Luck, 2014; Thigpen et al., 2017).

### Event-Related Potential Recording and Analysis

Electroencephalography (EEG) was recorded from 64 scalp sites using Ag/AgCl electrodes mounted on an elastic cap, according to the extended international 10–20 system (Neuroscan, Sterling, VA, United States), with the references on the left and right mastoids, and a ground electrode on the medial frontal aspect. Vertical electrooculograms (EOGs) were recorded supraorbitally and infraorbitally at the left eye. The horizontal EOG was recorded as the left versus the right orbital rim. EEG and EOG activity was amplified using a DC 100 Hz bandpass and continuously sampled in the 500 Hz/channel. The data was low pass filtered offline at 30 Hz (24 dB/oct), and all electrode impedances were maintained below 5 kΩ. Data pre-processing was performed in Neuroscan (Scan 4.4). An automated eye-movement correction program was used before artifact rejection (Gratton et al., 1983), based on the Least Mean Squares algorithm. ERP averages were computed off-line. Trials with remaining EOG artifacts (mean EOG voltage exceeding ± 80 mV), amplifier clipping artifacts, or peak-to-peak deflection exceeding ±80 mV, were excluded from averaging. The remaining epochs (mean: 163 epochs per body stimulus condition) were averaged separately for each participant and each body stimulus condition. ERP waveforms were time locked, and the average epoch was 1000 ms, including a 200 ms pre-stimulus baseline. The current study was the first to explore the electrophysiological characteristics of threat intention conveyed by body action, and thus we analyzed the brain information processing stream in the ERP results part.

Considering that this study was exploratory, a cluster-based permutation test was conducted using the FieldTrip toolbox (Oostenveld et al., 2011) to perform a planned comparison between pairs of conditions. The cluster-based permutation test uses non-parametric statistics to capture ERP effects without prior assumptions about their scalp distribution and latency range (Maris and Oostenveld, 2007). Each compete set of data was randomly divided into two subsets and a new summed *t*-value was calculated, which was repeated 10,000 times. The initial alpha value for cluster formation was set at alpha <0.01 to reduce the likelihood of large clusters spanning the entire dataset (Mensen and Khatami, 2013).

**FIGURE 2:**
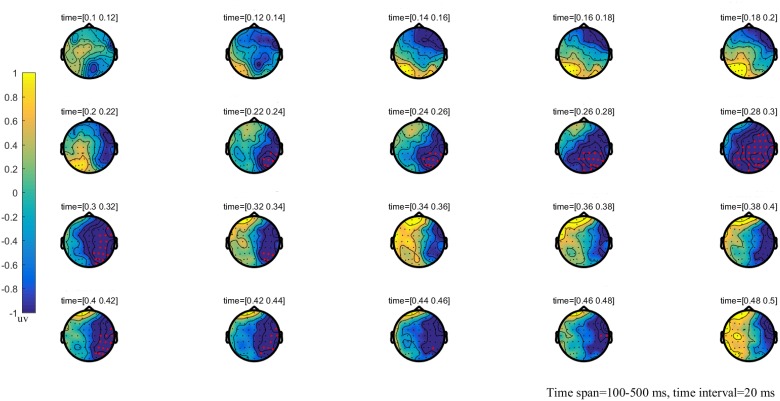
The global brain topographic map of the difference between NT and MT, time from 100 to 500 ms after stimulation onset. The results show that the difference of waveform between the two stimuli is mainly reflected in the right posterior brain area of 260–330 ms. Red points indicate that these electrodes are members of significant clusters.

**FIGURE 3:**
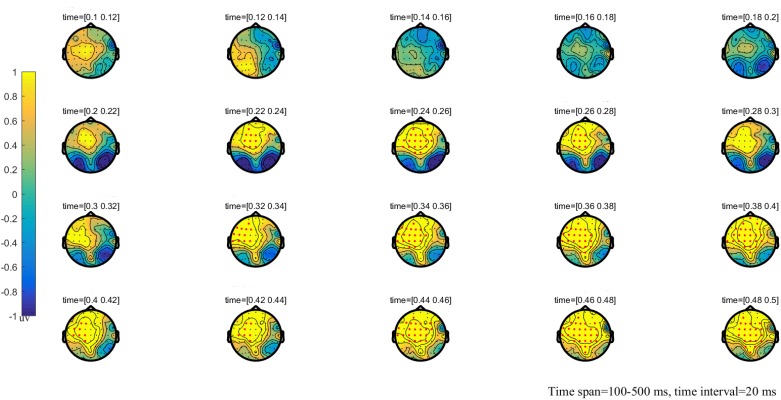
The global topographic map of the difference between NT and HT, time from 100 to 500 ms after stimulation onset. The results showed that the difference of waveform between the two stimuli is mainly reflected in the prefrontal area of 200–260 ms and the frontal-parietal and posterior area of 330–500 ms. Red points indicate that these electrodes are members of significant clusters.

**FIGURE 4:**
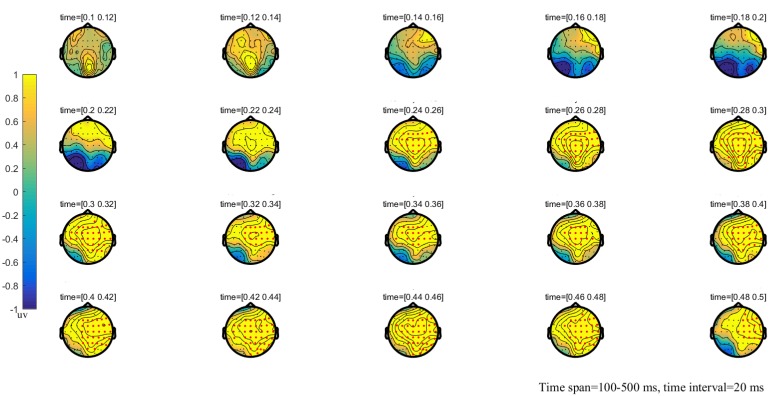
The global topographic map of the difference between MT and HT, time from 100 to 500 ms after stimulation onset. The results showed that the difference of waveform between the two stimuli is mainly reflected in the right posterior brain area of 260–330 ms and the frontal-parietal and posterior area of 330–500 ms after stimulation onset. Red points indicate that these electrodes are members of significant clusters.

**FIGURE 5:**
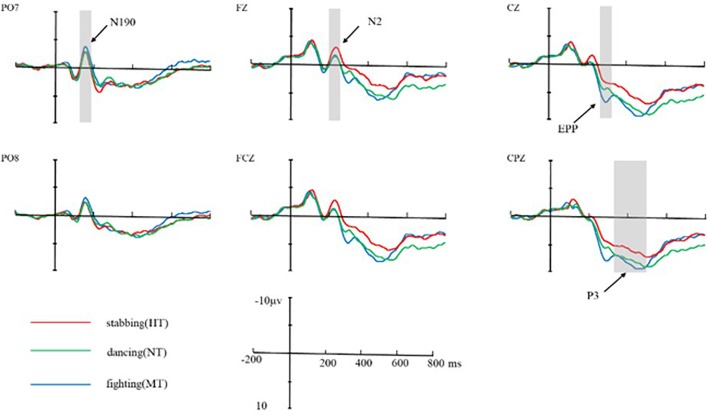
Average ERPs at Fz, FCZ, CZ, CPZ, PO7, and PO8 for HT, MT, and NT conditions. The neural activation (waves from at PO7 and PO8 for N190, Fz, FCZ for N2, CZ for EPP, and CPZ for P3) in contrast of HT condition, MT condition, and NT condition. Under N2 component, HT amplitude was larger than MT and NT; under EPP component, MT amplitude was larger than NT and HT.

**FIGURE 6:**
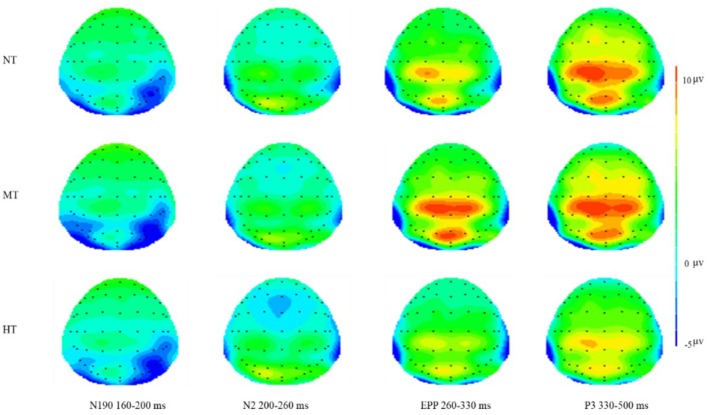
Topographical maps of three conditions on N190, N2, EPP, and P3. Significantly larger frontal N2 amplitude occurred in the HT condition and significant central EPP amplitude occurred in the MT condition.

As shown by the ERP’s grand averaged waveforms, according to the cluster permutation test (Figures 2–4), the ERPs elicited by the three conditions showed prominent differences from each other and these differences were the largest at the central and frontal sites (see Figures 5, 6). N2 over a frontal-central region across the scalp is attributed to attention resource for different negative pictures and threat detection (Thorpe et al., 1996). In addition, previous studies have shown that the anterior N2 component also represents the allocation of attention to novel or threatening stimuli (Yuan et al., 2007). During the later stages of stimulus presentation, P3, a positive-going centro-parietal ERP beginning approximately 300 ms after stimulus onset, was detected, reflecting cognitive evaluation of a stimulus’ meaning (Polich, 2007; Wei et al., 2016) and the elaborated processing of stimulus meaning (Hajcak and Olvet, 2008; Weinberg and Hajcak, 2010). Thus, we selected the following 12 electrode sites for statistical analysis: FZ, F3, F4, FC3, FC4, FCZ, C3, C4, CZ, CP3, CP4, CPZ (12 central-parietal and frontal sites) for EPP, and N2 and P3 according to the time and topography of these components; in addition, we selected PO7 and PO8 (two parietal-occipital sites) for N190 and EPN. Thierry et al. (2006) found a negative component peaking at 190 ms post-stimulus onset (N190), reflecting the structural visual encoding of bodies. At a later stage of visual processing, salient emotional bodies modulate the amplitude of the EPN, which reflects stimulus-driven attentional capture, in which relevant stimuli are selected for further processing (Schupp et al., 2004; Calvo and Beltrán, 2014). Thus, the N190 amplitude was quantified as the mean amplitude in a window of 160–200 ms of each target site. The mean amplitudes of N2 (200–260 ms), EPN (200–400 ms), EPP (260–330 ms), and P3 (330–500 ms) components were also analyzed using the above method.

The main aim of this study was to explore whether EPP is an electrophysiological indicator of intention processing, and whether this component would trigger an adaptive action response. A two-way repeated measure analysis of variance (ANOVA) was conducted for the amplitude of each component. ANOVA factors were threat intention (three levels: HT, MT, and NT) and electrode site (12 sites for N2, EPN, EPP and P3; two sites for N190). The dependent variable was the average amplitude of the time period. The significance level for all ANOVAs was set at 0.05. In all cases, these components were statistically evaluated using SPSS (version 20.0) and Greenhouse-Geisser correction was used when the hypothesis of sphericity was not met. For significant main effects or interactions, Bonferroni corrected *p*-values were reported for *post hoc* comparisons.

## Results

### Behavioral Results

The mean accuracy of three conditions was 96% (HT), 93% (MT), and 93% (NT), respectively. This showed that participants could perceive and classify the action intentions effectively. The results showed a main effect of threatening intention, *F*(2,52) = 17.123, *p* = 0.002, $\eta_{p}^{2}$ = 0.233, with significant faster reaction times following HT (*M* = 461 ms, *SD* = 33 ms) as compared with MT (*M* = 496 ms, *SD* = 26 ms, *p* < 0.001) and NT (*M* = 477 ms, *SD* = 37 ms, *p* = 0.003). A Bonferroni-corrected pairwise comparison showed that the difference between MT and NT was significant (Mean Difference = 19 ms, *SE* = 4.23, *p* = 0.019). The behavioral results showed that HT induced the fastest response, followed by NT, and the response induced by MT was slowest.

### Cluster-Based Permutation Test

The results of the cluster permutation test are shown in Figures 2–4, which revealed several significant clusters in different conditions. Note that significant clusters represent sampling points with spatial and temporal adjacency. The contrast between NT and MT elicited a significant negative cluster, with a time window during 260–330 ms typical of an EPP (sum-*T* = -424.86, *p* < 0.002). In the comparison between NT and HT, HT also showed an early cluster (from 200 to 260 ms; sum-*T* = 164.8, *p* = 0.002), which could be considered as an N2, and a significant positive cluster (330–500 ms; sum-*T* = 775.5, *p* ≤ 0.001), which could be considered as a P3. The significant cluster was a positive one exhibited in the MT/HT contrast during 260–330 ms (sum-*T* = 875.18, *p* = 0.018), which was identified as an EPP, and during 330–500 ms (sum-*T* = 775.5, *p* = 0.002), which was identified as a P3. The scalp distributions of these clusters informed our selection of several ERP components that fell within the clusters (see Figures 5, 6), which we investigated using ANOVAs. We also report ANOVAs on some components of *a priori* interest.

To summarize, statistically reliable N2, EPP, and P3 clusters were observed only in the comparisons among NT/MT/HT. HT exhibited a higher N2 and MT exhibited a higher EPP.

### ERP Analysis

We analyzed the brain information processing stream (including N190, N2, EPN, EPP, and P3) in the ERP results. As shown in Figures 5, 6, N190, N2, EPP, and P3 components were elicited by all three conditions. In this study, a significant main effect of threat intention was found for N190 [*F*(2,52) = 10.623, *p* = 0.003, $\eta_{p}^{2}$ = 0.422], N2 [*F*(2,52) = 15.623, *p* < 0.001, $\eta_{p}^{2}$ = 0.435], EPP [*F*(2,52) = 55.623, *p* < 0.001, $\eta_{p}^{2}$ = 0.435], and P3 [*F*(2,52) = 28.648, *p* = 0.006, $\eta_{p}^{2}$ = 0.222] amplitudes.

#### N190 Response

For the N190 response, mean amplitudes during the interval from 160 to 200 ms, a two-way repeated measures ANOVA was conducted for the amplitude of this component. ANOVA factors were threat intention (HT, MT, and NT) and electrode site (two sites, PO7, PO8). The results revealed that the main effect of intention processing was significant (*M*_HT_ = -2.520, *SD* = 0.861, *M*_NT_ = -2.514, *SD* = 0.933; *M*_MT_ = -3.278, *SD* = 0.870), *F*(2,52) = 10.623, *p* = 0.003, $\eta_{p}^{2}$ = 0.422. *Post hoc* analyses showed a significantly larger N190 amplitude in response to MT compared with HT (*p* < 0.01) and NT (*p* < 0.01). The interactions between threatening conditions and electrodes were not significant, *F*(2,52) = 1.317, *p* = 0.16. In addition, there was also no significant difference found between the NT and HT.

#### N2 Response

The potential component at 200–260 ms was analyzed through within-participant two-way ANOVA with intention (HT, MT, and NT) and electrode site (12 sites) This result revealed that main effect of threat intention was significant (*M*_HT_ = -1.17, *SD* = 0.563; *M*_NT_ = -0.635, *SD* = 0.647; *M*_MT_ = -0.622, *SD* = 0.570), *F*(2,52) = 15.623, *p* < 0.001, $\eta_{p}^{2}$ = 0.435. *Post hoc* analyses showed a significantly larger N2 amplitude in response to HT compared with all other stimuli (all *p* < 0.01). There was no significant difference found between the NT and MT (*p* = 0.099). Additionally, a main effect of amplitude at electrode sites was significant for N2 [*F*(11,286) = 255.75, *p* < 0.01, $\eta_{p}^{2}$ = 0.135]. The largest N2 amplitudes were recorded at the frontal electrode sites [e.g., Fz, F3, F4] and all anterior sites displayed a larger N2 than the posterior sites. The interactions between threatening conditions and electrodes was not significant, *F*(22,572) = 1.370, *p* = 0.56.

#### EPN Response

The potential component of EPN at 200–400 ms was analyzed using two-way ANOVA with threat intention (HT, MT, and NT) in PO7 and PO8 electrode sites. This result revealed no significant difference among three conditions, *F*(2,52) = 3.389, *p* = 0.078, $\eta_{p}^{2}$ = 0.122. No other comparisons were significant (all *p* > 0.69).

#### EPP Response (260–330 ms)

Event-related potential responses are shown in Figures 5, 6. The results of two-way ANOVA showed that the intention main effect was significant (*M*_HT_ = 0.848, *SD* = 0.453; *M*_NT_ = 1.998, *SD* = 0.443; *M*_MT_ = 3.811, *SD* = 0.580), *F*(2,52) = 15.623, *p* < 0.001, $\eta_{p}^{2}$ = 0.345. *Post hoc* analyses showed a significantly larger EPP amplitude in response to MT compared with the other kinds of stimuli (all *p* < 0.01). In addition, the amplitude of NT reached a more significantly positive deflection than HT (*p* = 0.002). Additionally, a main effect of amplitude at electrode sites was significant for EPP [*F*(11,286) = 342.29, *p* < 0.01, $\eta_{p}^{2}$ = 0.135]. The interaction between threatening conditions and electrodes was significant, *F*(22,572) = 9.123, *p* < 0.001.

#### P3 Response

For the P3 response amplitudes during the interval from 330 to 500 ms, the within-participant two-way ANOVA with threat intention (HT, MT, and NT) and electrode sites (12 sites) revealed that the intention main effect was significant (*M*_HT_ = 2.742, *SD* = 0.589, *M*_NT_ = 5.335, *SD* = 0.763; *M*_MT_ = 5.278, *SD* = 0.870), *F*(2,52) = 18.648, *p* = 0.0026, $\eta_{p}^{2}$ = 0.222. *Post hoc* analyses showed a significantly smaller P3 amplitude in response to HT compared with MT and NT (all *p* < 0.01). There was no significant difference found between the NT and MT (*p* = 0.164). Additionally, a main effect of amplitude at electrode sites was significant for the P3 component [*F*(11,286) = 179.34, *p* < 0.01, $\eta_{p}^{2}$ = 0.126]. The interaction between threatening conditions and electrodes was significant, *F*(22,572) = 5.154, *p* < 0.001.

#### Correlations

**FIGURE 7:**
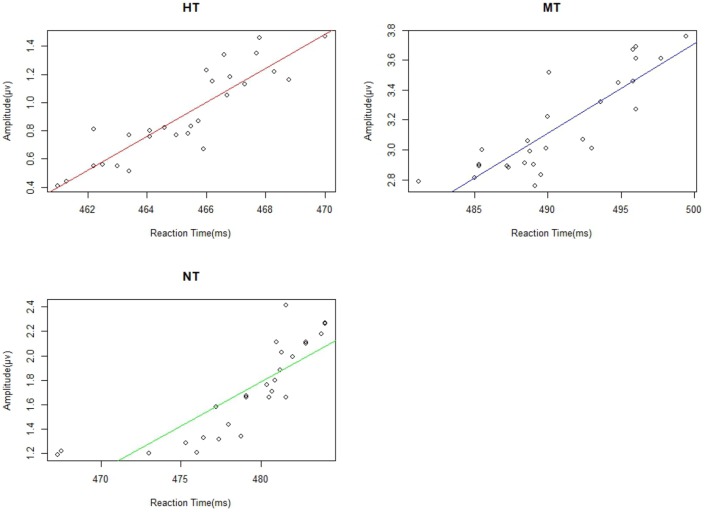
There were significant correlations between EPP and reactive time under different action conditions.

We calculated the correlations between reaction time and the amplitude of EPP, N190, N2, and P3 from the 27 participants. We found significant correlation between EPP amplitude and reaction time under HT (*r* = 0.787), NT (*r* = 0.671), and MT (*r* = 0.720) conditions (all *p*s < 0.001). Individuals with a lower EPP amplitude showed a faster response toward the threatening intention (Figure 7). The linear relationships among the RT (reaction time) with N190, N2, and P3 were also calculated; however, no significant correlation was found between them and the RT measure (all *p* > 0.10).

## Discussion

In previous studies on intention processing, researchers mainly used emotional body movements as the experiment object. However, because of the emotion and intention duality of emotional body movements, the perception and response of the human brain to intentions is always disturbed by emotions. Thus, we could not paint a conclusive picture about the relationship between intention processing and brain components. This study used threatening body actions without explicit emotions, but with obvious intentions, as experimental subjects. The behavioral results showed that participants responded faster to HT than to MT and NT stimuli, meanwhile, they responded faster to NT pictures than to MT pictures. More importantly, the amplitude of EPP in each condition was positively correlated with RT. Thus, EPP could be used an index of body intention processing of the brain.

At approximately 200–260 ms after stimulus onset, obvious N2 activity was generated at the frontal brain region in all three conditions, and larger N2 amplitudes were observed for the HT condition than for the MT and NT conditions over a frontal-central region across the scalp. N2 activation is indicative of attention resource for different negative pictures and threat detection (Thorpe et al., 1996). In addition, previous studies have shown that the anterior N2 component also represents the allocation of attention to novel or threatening stimuli (Yuan et al., 2007). Thus, our finding of larger N2 amplitudes in the HT condition is likely an index of a rapid attention allocation process that attends to threatening content (Li et al., 2005). Compared with the MT and NT pictures, the HT pictures often included salient threatening content (e.g., knife and gun), which has been shown to recruit human attention resources rapidly and automatically (Hansen and Hansen, 1988; Li et al., 2005). There was no significant difference between MT and NT. It is likely that these stimuli were just body actions. Accordingly, in the present study the detection of salient threatening information was facilitated.

Previous studies have shown that the perception of social intention is directly related to body actions; even for a simple gesture and its action, kinematics has shown differences between the conditions with and without communicative intentions (Pierno et al., 2008; Sartori et al., 2009). The purpose of our research was to explore the neural characteristics of perceiving intentions and the relationship between subsequent actions. The current results showed that there was a positive correlation between the amplitude of the EPP and the RT. The lower amplitude of EPP, shorter the RT. A possible explanation for EPP is that intention (i.e., carried by fighting or stabbing) can induce attention automatic allocation, which serves to understand action intention in the current task. In contrast to N2, the EPP component was significantly larger in the MT condition than in the HT and NT condition, which indicates that the action intention of the MT stimuli was detected and obtained more attention resources than other stimuli. This is most likely because, at the conscious level, the brain had begun to integrate the features for intention understanding. The intention of HT could be understood easily in the N2 stage because of salient stimuli, such as a gun or knife, and thus the EPP of HT was smaller than that of MT. The intention understanding of MT could be difficult because the solitary fighting action was ambiguous and not self-relevant. Previous studies have shown that threat bias was likely related to the direction of the threat in relation to the observer (Grèzes et al., 2013; de Valk et al., 2015). MT recruited more human attention resources for understanding the body intention, and thus, MT induced more positive drift compared with that of NT.

P3 signals the cognitive evaluation of a stimulus’ meaning (Polich, 2007; Wei et al., 2016). In the present study, HT stimuli evoked the smallest P3 amplitudes. The size of the P3 amplitudes in the present experiment may reflect the degree of an individuals’ decision-making. For a participant to make a correct behavioral response to the stimulus, all task-relevant information had to be evaluated correctly. It is possible that when the participants saw the gun or knife (in the N2 stage), they could identify easily that the picture should be attributed to high threat and there was no need to decode the intention of the body actions (in the EPP stage); therefore, the participants consumed fewer cognitive resources to make the decision. Therefore, it is easy to make the fastest decision under threat of the HT condition (with the least information burden), which would account for the small P3 amplitudes.

Many studies have reported a threat bias in which negative emotional body action or faces are prioritized over neutral (Hansen and Hansen, 1988; van Heijnsbergen et al., 2007; Feldmann-Wüstefeld et al., 2011; Borhani et al., 2015; de Valk et al., 2015). Our study verified those findings by showing whether perceiving explicit intentional actions could trigger faster actions or not. Our findings support the view of emotion and intention duality, and suggested that threat intention could foster action. In addition, MT stimuli triggered slower action than did NT, possibly because MT induces a complicated attention processing to intention, i.e., solitary and self-irrelevance rather than interaction and self-relevance. In other words, a stabbing picture could be perceived as a clear threat intention to the participants because a weapon or knife could assist them to understand the scene in the N2 stage; however, a solitary fight action might be more ambiguous as a source of threat intention when participants decode their intention in a limited time. Thus, we suggest that an explicit threat intention (e.g., HT) asks for immediate action and an ambiguous threat intention (e.g., MT) requires further exploration and hence needs more processing time and a somewhat slower response. Previous studies also support this view. For instance, Hortensius et al. (2016) aimed to explore the threat detection advantage of body stimuli. In their study, fearful/angry body stimuli were directed toward or away from the observer. Single-pulse transcranial magnetic stimulation to the left primary motor cortex was applied to measure motor evoked potentials from the right abductor pollicis brevis in response to angry and fearful bodily expressions, with blurred faces. The results showed that it was easier to recognize anger directed toward the observer than that directed away from the observer. The results provided direct evidence that clear threat intention could foster fast adaptive action. In the current study, fight actions (MT) promoted slower action because the human brain has to consume more attention resource to decode the ambiguous threatening intention. In addition, enhanced responses to dance action (NT) suggested that we can readily recognize the threat-unrelated but clear intention conveyed by body actions and make the correct decision. Thus, it is possible that different results may be obtained if MT stimuli become dual interaction or self-relevant, rather than solitary irrelevant threatening actions. Future studies could make use of our procedure with the aim of improving the ambiguous intention of solitary fighting actions. For example, one approach would be to present self-relevant or interpersonal, dynamic action pictures, not a static one.

In summary, the novel experimental design made it possible to directly test brain sensitivity to intention perception. The correlation between the amplitude characteristic of EPP and reaction time indicated that the EPP might be an index of intention processing of body actions. Furthermore, the clarity of intentional actions modulates the reaction time.

## Author Contributions

GW collected, analyzed, interpreted the data, and wrote the paper. JL and WN helped to analyze, interpreted the data, and helped to write the paper. GW and PW conceived the project. All authors read and approved the final version of the manuscript for submission.

## Conflict of Interest Statement

The authors declare that the research was conducted in the absence of any commercial or financial relationships that could be construed as a potential conflict of interest.

## Footnotes

1 https://www.wjx.cn/

2 https://www.gimp.org/
